# Glucose Oxidase, an Enzyme “Ferrari”: Its Structure, Function, Production and Properties in the Light of Various Industrial and Biotechnological Applications

**DOI:** 10.3390/biom12030472

**Published:** 2022-03-19

**Authors:** Jacob A. Bauer, Monika Zámocká, Juraj Majtán, Vladena Bauerová-Hlinková

**Affiliations:** 1 Institute of Molecular Biology, Slovak Academy of Sciences, 845 51 Bratislava, Slovakia; jacob.bauer@savba.sk (J.A.B.); monika.zamocka@savba.sk (M.Z.); juraj.majtan@savba.sk (J.M.); 2 Department of Microbiology, Faculty of Medicine, Slovak Medical University, Limbová 12, 833 03 Bratislava, Slovakia

**Keywords:** glucose oxidase, catalytic mechanism, GOx producing organisms, FAD binding domain, biosensors, nanosensors, antimicrobial effect

## Abstract

Glucose oxidase (GOx) is an important oxidoreductase enzyme with many important roles in biological processes. It is considered an “ideal enzyme” and is often called an oxidase “Ferrari” because of its fast mechanism of action, high stability and specificity. Glucose oxidase catalyzes the oxidation of β-d-glucose to d-glucono-δ-lactone and hydrogen peroxide in the presence of molecular oxygen. d-glucono-δ-lactone is sequentially hydrolyzed by lactonase to d-gluconic acid, and the resulting hydrogen peroxide is hydrolyzed by catalase to oxygen and water. GOx is presently known to be produced only by fungi and insects. The current main industrial producers of glucose oxidase are *Aspergillus* and *Penicillium.* An important property of GOx is its antimicrobial effect against various pathogens and its use in many industrial and medical areas. The aim of this review is to summarize the structure, function, production strains and biophysical and biochemical properties of GOx in light of its various industrial, biotechnological and medical applications.

## 1. Introduction

Glucose oxidase is an enzyme that has widespread applications in industry and biotechnology. Due to this, a deep understanding of its structure and function are warranted. Glucose degradation is the most universal metabolic process. In addition to its breakdown in glycolysis, glucose can also be directly oxidized to glucono-δ-lactone by a number of enzymes.

These fall into two classes: (1) the dehydrogenases glucose dehydrogenase (β-d-glucose: NAD(P)^+^ 1-oxidoreductase, E.C. 1.1.1.47) [1] and quinoprotein glucose dehydrogenase (d-glucose:ubiquinone oxidoreductase, E.C. 1.1.5.2) [2] and (2) the oxidases glucose oxidase (GOx; β-d-glucose:oxygen 1-oxidoreductase, E.C. 1.1.3.4) [3] and pyranose oxidase (pyranose:oxygen 2-oxidoreductase, E.C. 1.1.3.10) [4].

**Figure 1:**
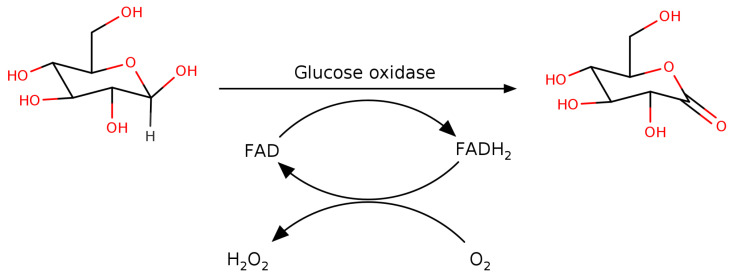
The general reaction of GOx.

The dehydrogenases oxidize glucose in one step using a co-factor, either nicotinamide adenine dinucleotide (phosphate) (NAD(P)${}^{+}$) or pyrroloquinoline quinone (PQQ), as the electron sink while the oxidases use a two-step mechanism in which a bound flavin adenine dinucleotide (FAD) co-factor is used to oxidize glucose to form glucono-δ-lactone and an enzyme-FADH${}_{2}$ intermediate followed by electron transfer to O${}_{2}$ to form H${}_{2}$O${}_{2}$ (Figure 1). The principal difference between GOx and pyranose oxidase is that the former is specific to β-d-glucose while the latter is also able to act on d-xylose, l-sorbose and d-galactose.

The high specificity, high turnover and high stability of GOx make it an ideal enzyme for biosensor applications [5], some of which will be described below. Although its rate constant is still several orders of magnitude below the diffusion limit, GOx has a much higher $k_{\mathrm{cat}}/K_{M}$ (on the order of $10^{6}$$M^{-1}\cdot s^{-1}$) compared with most other oxidoreductases, prompting at least one researcher to call it “the Ferrari of the oxidases” [6].

GOx is a member of the glucose-methanol-choline oxidoreductase (GMC oxidoreductase) superfamily. The members of this family are all FAD-dependent oxidoreductases that share a common fold [7,8]. They consist of two functional domains, an N-terminal FAD-binding domain, which contains a strictly conserved βαβ mononucleotide-binding motif and a more variable substrate binding-domain. As the name suggests, the members of this family oxidize a variety of substrates containing hydroxyl functional groups, including mono and di-saccharides, alcohols, cholesterol and choline. GOx is perhaps the most thoroughly characterized of these, and its mechanism will be described more thoroughly below.

## 2. Glucose Oxidase Structure

Glucose oxidase has widespread applications in industry and biotechnology. Due to this, a deep understanding of its structure and function are warranted. Perhaps surprisingly for such a well-studied enzyme, there are only seven crystal structures of glucose oxidase available, six from *A. niger* and one from *P. amagasakiense* [9,10,11,12]. The *A. niger* GOx structures range in resolution from 2.4–1.2 Å (Table 1); five of them are in hexagonal space groups (P$3_{1}21$ or P$3_{2}21$), and one is in an orthorhombic one (C22$2_{1}$). Although the active form in solution is a dimer, the asymmetric unit of each structure contains only monomer with the second monomer occupying a symmetry-related site.

These structures are all very similar, with a mean RMSD of 0.31 ± 0.06 Å over an RMSD range of 0.15–0.40 Å. The *P. amagasakiense* structure is unique in having a complete dimer in the asymmetric unit of space group P$2_{1}2_{1}2_{1}$. This is very similar to the *A. niger* GOx structures, with an average RMSD of 0.68 ± 0.04 Å over a range of 0.64–0.77 Å. As it has been the most thoroughly studied, *A. niger* GOx will be described below; however, because of the high structural similarity, much of the description of the overall structure will also apply for *P. amagasakiense* GOx.

### 2.1. Overall Structure

**Figure 2:**
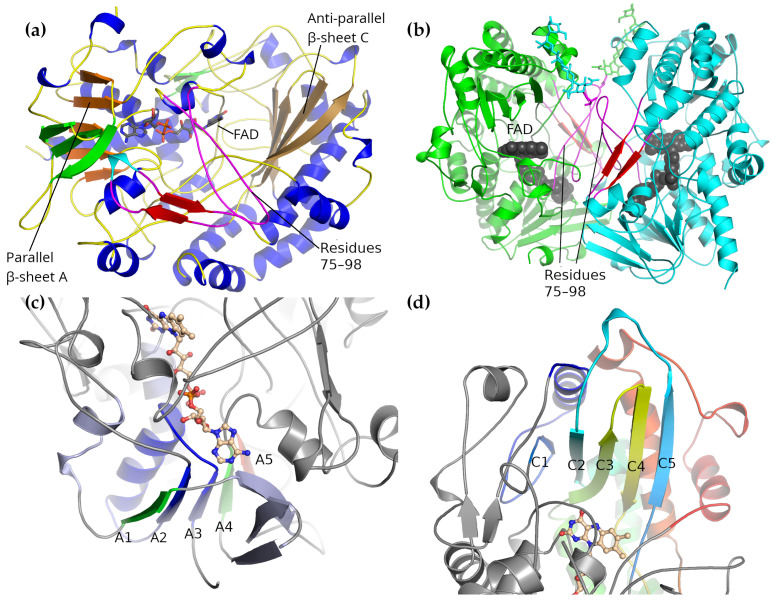
The overall structure of *A. niger* glucose oxidase. (**a**) The overall structure of the GOx monomer. The two central β-sheets are colored orange (parallel β-sheet A) and light brown (anti-parallel β-sheet C) and labeled. The two loops that participate in the dimer interface are colored magenta. (**b**) The GOx dimer. The two monomers are colored green and cyan, the FAD co-factor is shown as van der Waals spheres, the carbohydrate chains attached to Asn-89 that participate in the dimer interface are shown as sticks, and the loops that participate in the dimer interface are colored magenta. The more important of the two loops is labeled. (**c**) A closer view of parallel β-sheet A, which lies at the topological center of the enzyme. The β-sheet, together with the α-helix that forms part of the βαβ motif, is colored by residue from the N-terminus to the C-terminus based on sequence position to illustrate that all regions of the sequence take part in its formation. The α-helices and anti-parallel β-sheet that flank β-sheet A are shown in light blue. (**d**) A closer view of the anti-parallel β-sheet C, which forms a Greek-key motif. The residues surrounding β-sheet C form the second sub-domain and are colored by position from the N-terminal end to the C-terminal. The cytosolic-facing α-helices following β-strands C2 and C4 lie behind the central β-sheet in this view. All panels show the PDB structure 1cf3 [10].

The *A. niger* GOx monomer forms a rough, rounded rectangular prism shape, measuring roughly 60×52×37 Å. The monomer is comprised of a single folding domain with a rather complicated topology. When viewed in a manner that emphasizes the secondary structure elements while minimizing the contribution of the unstructured loops (as in Figure 2a,b), two subdomains can be seen, each one centered around a five-stranded β-sheet. These two subdomains can be associated with FAD-binding on the one hand and substrate binding on the other.

The first, FAD-binding domain is centered on a five-stranded parallel β-sheet (β-sheet A in Figure 2c), which is flanked by a smaller, three-stranded anti-parallel β-sheet on one side and three α-helices on the other. It contains the FAD-binding site and is similar to the FAD-binding motifs found in other FAD-binding, GMC oxidoreductase proteins, several of which are listed in Table 2. The most highly conserved part of this motif is the βαβ motif comprising strands A3, A4 and the α-helix between them; a ψ-BLAST search returns only local alignments to this region on the second iteration.

The parallel β-sheet at the center of this subdomain lies at the topological center of the molecule. Starting with strand A3, the chain traces out the βαβ motif (that is, A3–α-helix–A4) followed by a long loop forming part of the dimer interface and an extension forming about one-quarter of the second subdomain. The trace then returns to form strand A5. After A5, the chain forms a three-stranded anti-parallel β-sheet that forms part of the FAD-binding domain, and this is followed by strand A2. After A2 comes the remainder of the second subdomain followed by the last strand of the parallel β-sheet (A1). This is then followed by a long C-terminal α-helix.

In this context, it can be seen that the residues responsible for forming the FAD-binding pocket come from all parts of the polypeptide chain. The majority of the binding site is formed by residues from the N-terminal part of the sequence. Residues from the central region provide additional support, while most of the residues from the C-terminal region form the edges of the flavin binding pocket and also contribute two of the three active site residues.

The second subdomain is centered around a five-stranded anti-parallel β-sheet, which is supported by six α-helices (Figure 2d). The first of the five β-strands, C1, is much shorter than the others and arises from the extension following strand A4 of the FAD-binding domain. The remaining four strands are arranged in a Greek-key motif: strands C5 and C2 are connected through a loop that passes over strands C3 and C4, which are joined together by a hairpin loop.

Strands C2 and C3 are connected through a large excursion that forms three of the supporting α-helices. Following C4 is a long loop that forms the second part of the dimer interface followed by the last to helices of the subdomain. The six α-helices lie on the cytosolic side of the β-sheet, while the β-sheet itself forms one side of a deep pocket that has the active site and the flavin at the bottom.

Overall, this subdomain is similar to the binding sheet subdomain of cholesterol oxidase [26]. Several of the residues thought to be important for binding β-d-glucose are found here, including Trp-426, Phe-414 and Glu-412. The active site will be described more closely in Section 3 where the catalytic mechanism is described.

### 2.2. Dimer Interface

The *A. niger* GOx dimer measures approximately 60×52×77 Å. The FAD groups are located near the dimer interface but are more than 22 Å apart, making it unlikely that they communicate with one another through allostery (Figure 2b). The dimer interface is predominantly formed by residues from the long loop following strand A4 of the parallel β-sheet of the FAD-binding subdomain (residues 75–98) and from the loop following strand C4 of the anti-parallel β-sheet of the substrate-binding subdomain (residues 432–455). The loop containing residues 75–98 covers part of the FAD-binding pocket.

It was previously thought that the *apo* form of GOx was a monomer [27] and that FAD binding was coupled to dimer formation [28,29], and the position of this loop appeared to support those conclusions. More recent work has shown, however, that after the dissociation of FAD, the enzyme is not in a monomeric state but appears to form aggregates [30] and that the dimer does not dissociate during thermal denaturation [31]. GOx does appear to dissociate into monomers at pH 5 and below in the presence of sodium *n*-dodecyl sulfate [32].

In addition to forming part of the FAD-binding site, residues 75–98 also from part of the wall of the active site cavity of the second monomer in the dimer: the complete dimer is needed to properly assemble a complete active site cavity, which may account at least in part for the dimeric form of the holoenzyme [27,33].

### 2.3. Active Site

**Figure 3:**
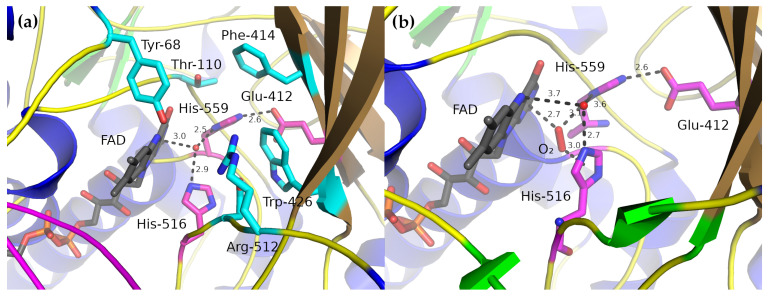
The active site of *A. niger* GOx. (**a**) The active site of the oxidized form of the enzyme in the absence of any substrate. The residues potentially important for catalytic activity are colored magenta; those which are thought to be involved in binding β-d-glucose are colored cyan. The water molecule near the center is thought to indicate the approximate location of the β-d-glucose O1 hydroxyl in the substrate-bound conformation. PDB structure 1cf3 [10] is shown. (**b**) The active site of an engineered GOx mutant showing an O${}_{2}$ molecule bound to the active site (PDB structure 5nit from Petrović et al. [12]).

In the complete dimer, the active site lies at the bottom of a deep, cone-shaped cavity whose apex is centered on the N5 atom of the middle flavin ring, the one that accepts the hydride during reduction. Only three amino-acid side-chains are near this center, His-516, His-559, and Glu-412 (Figure 3). His-559 is fixed by a strong hydrogen-bond to Glu-412 and Glu-412 is largely fixed by the side-chains of the surrounding residues (Ala-349, Phe-351, Phe-414, Trp-426 and Leu-428). The side-chain of His-516 is less constrained and is more conformationally flexible [12].

As all these structures were produced from crystals grown at pH 5.1–6.9, it seems likely that both histidine residues are protonated on both N atoms. All but one of the GOx crystal structures also have a water molecule at the center of the active site, forming hydrogen-bonds to both His-516 and His-559 and lying 3.0 Å away from the N1 ring of FAD (Figure 3a). The location of this water molecule has been taken to represent the likely position of the O1 hydroxyl group of β-d-glucose [10]. Kinetic, structural and thermodynamic data have allowed likely roles to be assigned to these residues.

Despite numerous attempts, no structure of GOx with a bound substrate has been determined [10,12]. Wohlfahrt et al. [10] used manual docking and molecular dynamics simulation to characterize a likely enzyme–substrate complex using oxidized *A. niger* GOx and β-d-glucose. Their modeling suggested that the residues most important for the enzyme–substrate complex included the catalytic His-516 and His-559 along with Tyr-68, Thr-110, Phe-414, Trp-426, Arg-512 and Asn-514. His-516 and His-559 formed hydrogen-bonds directly with the O1 hydroxyl group, which is expected to lose a proton in the catalytic step, while Asn-514, Arg-512, Tyr-68 and O4 of the FAD all form hydrogen-bonds to the remaining glucose hydroxyl groups, thereby, anchoring the substrate in position.

## 3. Catalytic Mechanism

The catalytic mechanism of GOx has been studied for many years, primarily using kinetic methods [34]. These, together with the enzyme structure, provide the identities of the active-site residues and allow a description of the mechanism of the reaction to be proposed. Many many different substrates and electron-acceptors have been employed (see Table 3); however, we will concentrate on the “natural” reaction using β-d-glucose as the substrate and O${}_{2}$ as the electron acceptor.

**Figure 4:**
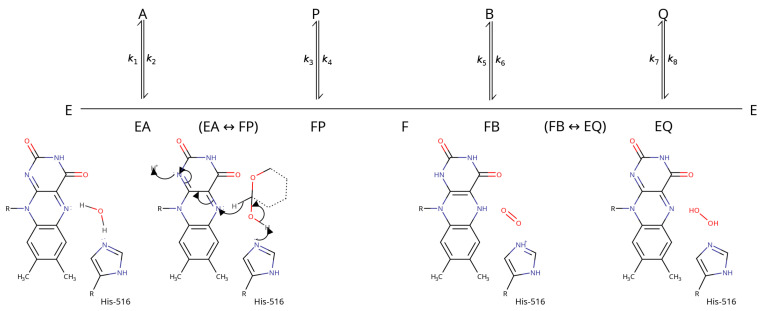
A scheme illustrating the overall glucose oxidase reaction. A indicates glucose, P indicates glucono-δ-lactone, B represents O${}_{2}$, Q represents H${}_{2}$O${}_{2}$, E represents the oxidized form of GOx bound to FAD, and F stands for the reduced form of GOx bound to FADH${}_{2}$. The individual components of the reaction are illustrated below the scheme; from left to right they are reduced GOx (E), the most likely mechanism of the GOx reduction half-reaction (EA ↔ FP), the GOx–O${}_{2}$ complex (FB) and the GOx–H${}_{2}$O${}_{2}$ complex (EQ).

GOx operates using a Ping-Pong Bi Bi mechanism in which β-d-glucose oxidation and O${}_{2}$ reduction occur in two different steps [35,39,40,41] (Figure 4). This allows the oxidation and reduction half reactions to be analyzed independently using steady state kinetics. pH profiling showed that the p$K_{a}$ of the amino-acid side-chains in the active site of the enzyme–substrate complex is between 6.9–7.8 [35,42].

Several lines of evidence ascribed this p$K_{a}$ value to His-516 [40,41]. The most likely mechanism involves a base-catalyzed hydride transfer from the glucose C1 to the flavin N5. Glucose binding is thought to displace the water molecule observed in the active site of the GOx crystal structures, which abstracts a proton from His-516 as it leaves (this abstraction appears to be necessary, according to modeling studies, because His-516 cannot adopt a proper catalytic position when doubly-protonated) [10].

Hydride transfer occurs in a concerted step in which a proton is removed from the glucose O1 hydroxyl group by a basic group on the enzyme while a hydride is transferred from the glucose C1 to the flavin N5 [43]. The kinetic rate constants associated with this step show a large H/D kinetic isotope effect, suggesting that this transfer is the rate-limiting step of the reductive half-reaction [44]. The basic group on the enzyme is likely to be His-516: a His-516-Ala mutant had practically no catalytic activity [41].

Considerations of resonance suggest that hydride transfer should create a negative charge around the FAD N1 atom, which have been confirmed by NMR studies of GOx at pH 5.6 in the absence of oxygen [45]. Following the reduction half-reaction, the enzyme is left in a reduced form with a bound glucono-δ-lactone. The product is then displaced by water or possibly O${}_{2}$ and the oxidation half-reaction follows.

The kinetic and thermodynamic parameters for the oxygen-binding step suggest that O${}_{2}$ easily and rapidly diffuses into the enzyme [35,44,46]. Indeed, structural studies on mutant GOx enzymes engineered to be more stable and have greater catalytic efficiency found O${}_{2}$ bound in the active site [12] (Figure 3). The oxygen bound in a small pocket formed by shifting His-516 up towards the active-site opening and displacing the active-site water molecule so that it lay outside hydrogen-bonding range of FAD (its closest approach was 3.7 Å from the N5 atom of FAD).

In its ground state, molecular oxygen is a paramagnetic triplet, making its insertion into diamagnetic organic molecules a spin-forbidden process [47]. In the active site, one of the O${}_{2}$ atoms is 2.7 Å away from the N1 flavin ring while the second is 3.0 Å away from the His-516 ring. This position led Petrović et al. [12] to suggest that the oxidative half-reaction might rely on orbital coupling between the oxygen and the π electrons in the His-516 side-chain to overcome this limitation.

Generally, O${}_{2}$ oxidizes organic substrates by transferring electrons one at a time, forming free-radical intermediates, and most analyses of of the oxidative half-reaction assumed a step-wise electron transfer [34,48]. The rate-limiting step appears to be the transfer of the first electron from flavin to O${}_{2}$ to produce the flavin semiquinone radical and the superoxide anion [41]. Kinetic isotope effect experiments show that no proton transfer takes place during this step [35,44]. The second electron transfer step, from the flavin semiquinone radical to the superoxide anion, happens quickly, with a second-order rate constant of $10^{9}$ M${}^{-1}\cdot$ s${}^{-1}$ [49].

### Glycosylation

Consistent with its role as an extracellular protein, GOx is glycosylated, with carbohydrates, mostly mannose-like sugars, comprising between 10 and 16% of its final molecular weight [27,36,50]. Both *N*-linked and *O*-linked sugar chains are present. The removal of 95% of the GOx carbohydrate content had a noticeable effect on the kinetics of glucose oxidation, its stability at low pH and the number of available isoelectric forms but did not affect its thermal stability or the optimal pH and temperature of the catalytic reaction [50,51].

More particularly, a study using H/D isotope substitution found that reduced glycosylation appeared to lower the enthalpy of activation and, therefore, increase the activity of the enzyme [52]. Deglycosylation typically removes all of the *O*-linked sugars at their linkage to the amino-acid side chain but leaves the first *N*-acetylglucosamine (Nag), potentially allowing those sites to be identified in the crystal structure [53].

Potential glycosylation sites can be identified by the consensus sequence N-X-T/S, where X is any amino-acid residue except proline [54]. Eight such sites are present in *A. niger* GOx (residues Asn-43, 89, 161, 168, 258, 355, 388 and 473); seven of these sites have been confirmed in at least one of the known *A. niger* GOx structures. Asn-43 does not appear to be glycosylated in any of these structures, and peptide sequencing work also suggested that no carbohydrate is attached here [54].

## 4. Natural Sources of Glucose Oxidase

GOx is primarily produced by fungi and insects [55]. There were early reports of GOx activity in extracts from red algae [56], citrus fruits [57], mammalian tissues [58] and bacteria [59,60,61,62]. A careful examination of these studies suggests that the activities from plants and mammals are not due to GOx and that several of the activities reported for bacteria appear to be due to the NAD-dependent glucose dehydrogenase. Two early studies on extracts from *Burkholderia pseudomallei* [59] and *Acetobacter suboxydans* [60] may be due to GOx; however, there were no later studies confirming this.

All these studies made use of what would now be considered rather crude cell-free preparations, which would make it difficult to distinguish between the glucose oxidase activity of GOx and that of pyranose oxidase. To date, there appears to have been no confirmed glucose 1-oxidase identified in any bacterial strain, and a recent phylogenomic study of the GMC oxidases found that pyranose oxidase is only one of the GMC proteins present in both fungi and bacteria [8].

To unambiguously identify those organisms that are presently known or suspected of harboring GOx, the NCBI Protein database, UniProtKB and the Brenda Enzyme Database were searched for entries classified as having E.C. number 1.1.3.4, and some of the recent literature was surveyed. After discarding duplicate entries and removing those that were mis-annotated (for example, a *Streptomyces coelicolor* alditol oxidase, E.C. 1.1.3.41, was incorrectly classified as GOx in one of these databases), a total of 50 unique species were identified; 45 species were from fungi, and five were from insects.

The insect species include *Apis melifera* (the honey bee) [63], *Helicoverpa armigera* (Cotton bollworm) [64], *Heliothis viriplaca* (marbled clover, a moth), *Mythimna separata* (Oriental armyworm) and *Spodoptera exigua* (Beet armyworm) [65]. No mammalian, plant, or bacterial species were found. The results are listed in Table 4.

Only 27 of the 50 species had sequences available in one of these three databases. Table 4 shows the similarities of these sequences to *A. niger* GOx according to BLAST. All sequences are roughly the same length (generally around 600 residues) and the alignment covers at least 89% of the *A. niger* sequence. They can be divided into three main groups: The two *Aspergillus* species share 85% identity, next are the *Penicillium* and *Talaromyces* species with 63–67% identity to *A. niger* (with one exception), and last are all other species, which have only 27–35% identity.

As shown in Table 2 above, a sequence similarity of this is generally the same identity shared between *A. niger* GOx and other, functionally non-equivalent GMC oxidases. This means that it will generally not be possible to identify as GOx uncharacterized sequences from more distantly related organisms even within the same kingdom using BLAST searches alone.

**Figure 5:**
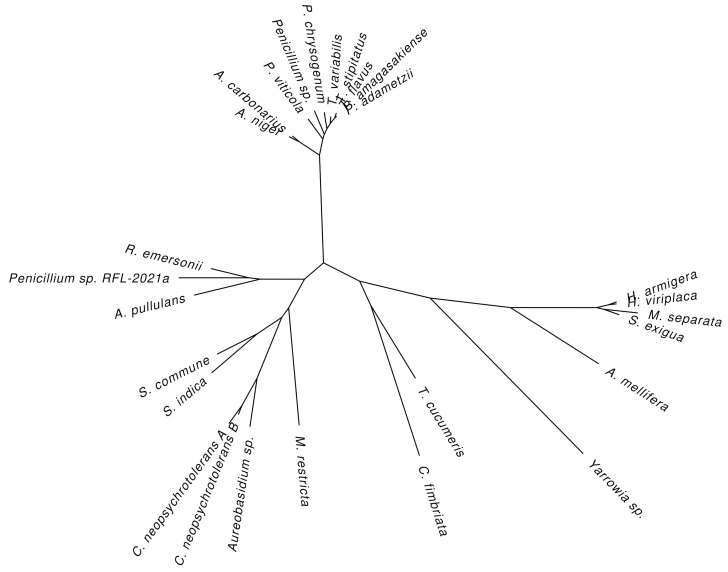
A phylogenetic tree based on an alignment of all sequences given in Table 4. The separation between the insect and fungal GOxes can clearly be seen. Within the fungal group, the *Aspergillus, Talaromyces,* and most of the *Penicillium* species cluster in their own clade, while the other species cluster in a different one. This tree was generated by PhyML [91] based on an alignment prepared by Clustal Ω [92].

A phylogenetic tree based on an alignment of all these sequences generally maintains the grouping seen in Table 4, putting the insect GOxes together in one branch and separating the fungal GOxes into two separate branches (Figure 5). One branch clusters the *Aspergillus, Talaromyces,* and most of the *Penicillium* species, while the other branch contains all the remaining species. Curiously, the *Yarrowia* sp., *Thanatephorus cucumeris,* and *Ceratocystis fimbriata* species are closer to the insect species than the fungal species.

**Figure 6:**
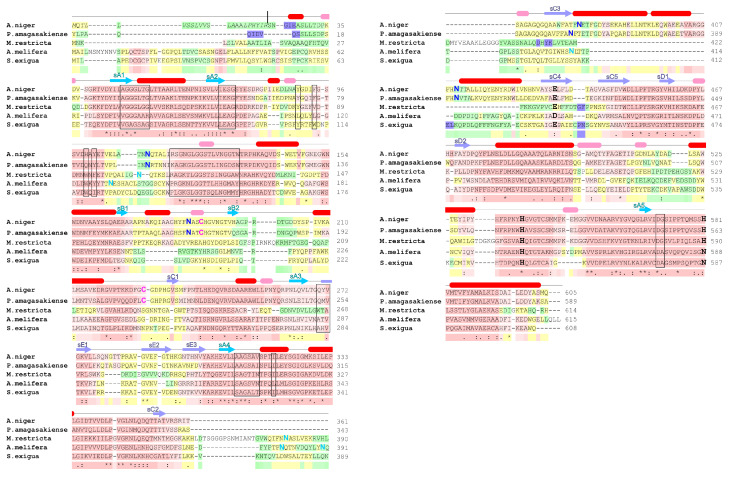
Amino-acid sequence alignment of GOx from *A. niger* (UniProt ID P13006), *P. amagasakiense* (P81156), *M. restricta* (A0A3G2SBT7), *A. melifera* (Q9U8X6) and *S. exigua* (D9ZFI1) performed by t-coffee [93]. The overall amino-acid sequence identity is 36%. The alignment is over the full-length sequences found in the UniProt database, which includes likely N-terminal presequences. The secondary structure elements shown are based on those derived from the 1.9 Å wild-type *A. niger* GOx structure (PDB ID 1cf3). Red and pink cylinders represent α-helices and $3_{10}$ helices, respectively; blue and purple arrows indicate parallel and anti-parallel β-sheets, respectively. Individual β-strands within each β-sheet (A–E) are numbered according to their position in the structure rather than the sequence (see Figure 2). The *A. niger* GOx presequence cleavage site (R22) is marked with a vertical line. The positions of the crucial catalytic residues His-516, His-559 and Glu-412 (corresponding respectively to residues 538, 581 and 434 in the full-length sequence) are shown in larger and bold-face type. Residues participating in FAD binding are in boxed. Cysteines forming a disulfide bond in the *A. niger* and *P. amagasakiense* structures are shown in larger, bold-face, pink type. The glycosylation sites observed in the *A. niger* and *P. amagasakiense* structures (Asn-89, 161, 355 and 388, corresponding respectively to residues 111, 183, 377 and 410 in the full-length sequence) are shown in larger, deep blue type. The positions of potentially glycosylated asparagines in the *M. restricta*, *A. melifera* and *S. exigua* GOx sequences are shown in larger, light blue type.

A multiple-sequence alignment of three fungi (*A. niger*, *P. amagasakiense* and *M. restricta*) and two insect (*A. melifera* and *S. exigua*) GOx sequences highlights the conserved areas (Figure 6). The overall sequence identity relative to the consensus sequence reached approximately 37%; however, there is a clear division between the fungal (43%) and insect (28%) sequence groups (see also Figure 5).

The largest differences occur at the N-terminus where signaling pre-sequences may be located. Overall, the alignment shows several large blocks of relatively conserved residues separated by regions of low identity. As described above (Section 2.1), the residues forming the FAD binding site are distributed throughout the N-terminal, central and C-terminal part of the molecule, and the residues involved in FAD binding all occur in one of these relatively conserved blocks.

Outside the N-terminus, the regions of lowest identity correspond to residues 83–93, 145–189, 340–408 and 490–509 in the *A. niger* GOx structure. Residues 145–189 form part of the GOx outer surface distant from the active site; however, the remaining three appear to be structurally important. Residues 83–93 form an important part of the dimer interface and the insect sequences have a five-residue insertion in this area. Residues 490–509 occur near the dimer interface and also feature a five-residue insertion in the insect and *M. restricta* sequences. This region is also in contact with the loop containing residues 83–93, so these insertions may be correlated. Finally, residues 340–408 include the loop between β-strands C5 and C2 and the extension following C2.

The *M. restricta* and insect sequences have a poorly-conserved insertion between β-strands C5 and C2 and are lacking the sequences that comprise the either just the first two (insect sequences) or all three (*M. restricta*) of the α-helices between C2 and C3. Generally, the catalytic residues His-516, His-559 and Glu-412 are conserved, although only His-516 is absolutely conserved in all five sequences.

In the insect sequences, His-559 is replaced with an asparagine (a substitution that is not uncommon in the GMC family in this position generally), while Glu-412 is replaced with an aspartate in *A. melifera*. These substitutions suggest that there are subtle differences in the conformation of the active sites in these proteins; they also provide additional support to the designation of His-516 as the primary catalytic residue as described in Section 3.

The sequence alignment also shows that the positions of Cys-164 and Cys-206, which form disulfide bonds in the *A. niger* and *P. amagasakiense* structures, are conserved only in these two sequences. The cysteines in *M. restricta*, *A. melifera* and *S. exigua* appear in different locations in the sequence, suggesting either differences in disulfide bond formation or no disulfide bonds occur in these proteins.

Finally, it may be worth noting that the *A. niger* and *P. amagasakiense* glycosylation sites identified structurally (Asn-89, 161, 355 and 388 in the *A. niger* 1cf3 structure corresponding to Asn-111, 183, 377 and 410 in Figure 6) do not appear to be generally conserved. Only an equivalent to Asn-89 appeared in the same general location in *M. restricta* and *A. melifera* GOx. The positions of the remaining potential glycosylation sites are either shifted or missing in the other three GOx sequences, suggesting alternative patterns of glycosylation. In particular, the alignment in Figure 6 shows that *M. restricta* and *A. melifera* do have potential N-X-(T/S) glycosylation motifs in the neighborhood of residues Asn-355 and Asn-388, suggesting that at least one glycosylation site might be expected in this location.

## 5. Industrial and Medical Applications of GOx

GOx is used in many branches of industry because of its ability to oxidize glucose and produce hydrogen peroxide. Its rapid turnover and high stability finds it many applications in the food, pharmaceutical, medical, textile and power industries. For many of these applications GOx is used in a biosensor or nanosensor [94,95,96,97,98,99], in nanoparticles [100,101,102] or in nanosheets [103].

In many modern applications, GOx is often used in combination with other enzymes, for example, tyrosinase in the analysis and discrimination of musts and wines [104], α-amylases and xylanases for improving the quality of dough and bread [105], peroxidase for accurately measuring the level of glucose in blood and saliva [106] and tears [94], the autophagy inhibitor chloroquine in cancer intervention therapy [101] and insulin for regulating blood glucose levels in diabetes [107,108]. Finally, it has been combined with the anti-cancer drug tirapazamine and human serum albumin to create a nanoreactor capable of increasing the levels of hypoxia and reactive oxygen species and inhibiting tumor growth [109].

The applicability of GOx primarily depends on its quantity, thermal stability and activity. Many studies focused on identifying which fungal strains are better for biosensor development, which are better for clinical studies and which are better for biochemical diagnostic tests [55]. Optimal GOx utilization also requires consideration of the type of matrix on which GOx is bound and type of media and conditions under which it is used. Consequently, in addition to identifying the best GOx producers and the ideal conditions for its stability and activity, the development of different binding materials, environmental conditions and detection systems are also very important for expanding the range of GOx’s industrial applications.

### 5.1. Industrial Production of GOx

Industrially, the most important producers of GOx are species belonging to the genera *Aspergillus* and *Penicillium* [55,110,111], especially *A. niger* and *P. amagasakiense*. These genera are important because of their metabolic versatility and because they are “generally regarded as safe” by regulatory agencies [112].

Overall, *P. amagasakiense* GOx is catalytically more effective than *A. niger* GOx, with a 6× lower Michaelis constant ($K_{m}$) for β-d-glucose and 10× higher catalytic efficiency ($k_{\mathrm{cat}}/K_{m}$) [113]; however, it is also less stable and has a lower antimicrobial activity [114]. This prompted at least one group of researchers to create a hybrid GOx with improved stability and catalytic efficiency by genetically combining elements from both *A. niger* and *P. amagasakiense* GOxes [115]. Other researchers have attempted to improve the antioxidant capabilities [116] and thermal stability [117] of *A. niger* GOx.

Fungal GOx is produced by solid state fermentation (SSF) and submerged fermentation (SmF) [118,119]. Submerged fermentation is the more useful method because environmental factors can be controlled more easily. A study on the variability of GOx from *Aspergillus tubingensis* CTM507 produced by SSF and SmF found that SmF produced GOx more efficiently but that the GOx produced by SSF had a higher activity (170 U mL${}^{-1}$ for SSF versus 43.73 U mL${}^{-1}$ for SmF) [120]. Both of these methods have limited capabilities, so more efficient ways of producing GOx are being researched using genetic recombination techniques, mutagenesis and immobilization methods [3,55].

Glucose and saccharose are the carbon sources most often used for the industrial production of GOx [3,121]. GOx production can be induced from *A. niger* by glucose [121], CaCO${}_{3}$ [122], Mn${}^{2+}$, Co${}^{2+}$, thioglycolic acid, gluconic acid [123], EDTA (ethylene diamine tetra-acetic acid) Zn${}^{2+}$ and Fe${}^{2+}$ [116]. Inhibitors of *A. niger* GOx include the Ag${}^{+}$, Hg${}^{2+}$, Cu${}^{2+}$ and Mg${}^{2+}$ ions, CaCl${}_{2}$ [124,125], hydrogen peroxide accumulation [116], arsenates, *p*-chloro-mercapto-benzoate, phenyl mercuric acetate [125], hydroxylamine, hydrazine, phenyl hydrazine, dimedone, NaHSO${}_{4}$ [126], guanidinium chloride, urea and SDS (sodium dodecyl sulfate) [116].

The low fermentation capacity, complicated purification process and effectiveness of GOx limit the applicability of GOx from natural sources. Attempts to improve GOx production through the mutagenesis and screening of production strains has enhanced GOx production in *A. niger* by about 77% [127]. Genetic recombination has also been used to express GOx heterologously. The most effective cloning techniques and highest overexpression levels were reported for *Saccharomyces cerevisiae* and *Escherichia coli* and other *Aspergillus* and *Penicillium* species have also been used as hosts [128,129,130].

Other candidates for the industrial production of GOx are *H. polymorpha* and *P. pastoris* [131,132]. Along with *S. cerevisiae*, these yeasts have the advantages of rapid growth and extracellular protein production; the highest level of *A. niger* GOx production from *S. cerevisiae* is currently 9 g L${}^{-1}$. Unfortunately, GOx produced by *S. cerevisiae* and *H. polymorpha* tend to produce over-glycosylated forms of GOx with reduced activities [133]. However, *P. pastoris* has been successfully used for the production of a GOx from *A. niger* and *P. variable* P16, which was not over-glycosylated [134,135].

### 5.2. Use of Glucose Oxidase in the Food Industry

GOx has several important applications in the food industry [55], including the baking industry, the production of drinks, the production of gluconic acid and food preservation. In the baking industry, GOx is used as an oxidant to improve the quality of bakery products [136,137,138]. The hydrogen peroxide produced by GOx makes the dough more elastic and viscous [139] and lipase and GOx can increase the quality and durability of bread [140].

Generally, bread quality depends on wheat quality and dough rheology depends on the quantity of enzymes added [141,142]. GOx causes the formation of protein fibers in dough. Basal additives, such as GOx, ascorbic acid and α-amylases decrease bread fragility and enhance chewability, adhesion, elasticity and cohesion [143]. The addition of fungal xylanase has a positive effect on flours and crumb firmness [105,144].

GOx also finds use in the reduction of the alcohol content of wine. Warmer temperatures during the growth season are likely to increase the level of glucose in wine grapes. By decreasing the level of glucose, which would otherwise be transformed into alcohol through anaerobic fermentation, GOx could decrease the quantity of alcohol in the resulting wine [145]. The hydrogen peroxide produced by GOx has a bactericidal effect on the corrosive acidic and dairy microbes produced during the fermentation process. The hydrogen peroxide produced can be removed by the enzyme catalase, which transforms H${}_{2}$O${}_{2}$ into oxygen and water. GOx in combination with catalase decreases the quantity of alcohol better than GOx alone by converting glucose into gluconic acid [146].

For measuring the quantity of glucose in liquids, GOx has also been incorporated into analytical devices known as biosensors. These devices combine a biological component, frequently some kind of enzyme or antibody, with a physical or electrical transducer and an electrical component to provide a measure of the analyte of interest [147]. For example, Garcia-Hernandez et al. [104] constructed a bioelectronic “tongue” consisting of enzymes (tyrosinase and glucose oxidase) and polypyrrole or polypyrrole/Au nanoparticles to measure the alcoholic degree in musts and wine.

The information from this sensor can help to predict the characteristics of the finished wine at the beginning of the vinification process. Lopes et al. [148] created a biosensor that consisted of GOx and immobilized horseradish peroxidase to determine the quantity of glucose in beverages, such as orange juice and energy drinks. Finally, Mason, Longo and Scampicchio [149] created an electrochemical biosensor that consisted of GOx immobilized on a nylon nano-fiber membrane to determine the level of glucose in brewed beer.

Such devices also allow the level of glucose to be measured in “sugar-free” foods intended for diabetics. For example, Kalaivani et al. [98] constructed a reliable sensor for detecting nanomolar amounts of glucose in food products.

Its production of gluconic acid also finds some uses for GOx in the food industry. Gluconic acid is used as an acid regulator, color stabilizer, antioxidant and chelating agent in food and drinks [150]. Gluconic acid is also applied in the dairy industry in cheese curd production, improving thermal stability of milk and cleaning of aluminum tins. The most popular application of gluconic acid in the food industry is its use as an acid regulator and antioxidant [151].

By removing oxygen and glucose from food, GOx can be used to lengthen shelf-life through two reasons. First, during food preservation, an unwanted reaction may occur between the nuceophilic functional groups on amino acids with the active carbonyls on sugars to cause non-enzymatic browning through the Maillard reaction. By removing unwanted sugars from the food to be preserved, this browning is prevented.

For example, using GOx to remove glucose residues from dried eggs improves their durability [152]. The hydrogen peroxide produced during this reaction also helps to destroy all pathogen microbes potentially present in raw eggs. Extra hydrogen peroxide can then be removed by the enzyme catalase [153]. The combination of GOx and catalase can also be used to control the non-enzymatic browning of fruit and tomato paste.

The second reason is that the extra oxygen can support bacterial growth, and removing the excess oxygen will inhibit the growth of aerobic bacteria. The removal of excessive oxygen is essential for canned foods [154] and Karimi et al. [155] studied the removal of dissolved oxygen in water through its reduction by glucose, catalyzed by glucose oxidase and catalase. GOx can also be used to remove of oxygen from canned drinks (such as beer and wine) to help them keep their color and taste [156,157]. The decomposition of mayonnaise is connected with lipid peroxidase [158] and GOx and catalase can slow down lipid peroxidation: oxygen removed during glucose reduction will not be available for lipid metabolism.

GOx is also used directly as an antimicrobial agent in the food industry. It was demonstrated to decrease the growth of many pathogenic bacteria, including *Clostridium perfringens, Campylobacter jejuni, Salmonella infantis, Staphylococcus aureus* and *Listeria monocytogenes* [159]. Vartiainen, Ratto and Paulussen [160] found that immobilized GOx inhibits the growth of *Escherichia coli* and *Bacillus subtilis* and Malherbe et al. [130] demonstrated that *S. cerevisiae* producing GOx from *A. niger* had antimicrobial activity against bacteria that produce lactic acid and acetic acid.

Polyamide and ionomer films with immobilized GOx inhibited the growth of *E. coli* CNCTC 6859, *Pseudomonas fluorescens* CNCTC 5793, *Lactobacillus helveticus* CH-1, *Listeria ivanovii* CCM 5884 and *Listeria innocua* CCM 4030 on agar medium [161]. Yuan et al. [162] developed a photodynamic antimicrobial system consisting of glucose, GOx and horseradish peroxidase to inactivate bacterial and fungal pathogens. Finally, Xu et al. [163] developed a biosensor comprising of antibodies, GOx, gold nanoparticles, shell magnetic beads, polydopamine and polymeric nanocomposites for the detection of pathogens in foods. This biosensor was able to detect *E. coli* O157:H7 with a detection limit of 10${}^{2}$ cfu/mL.

### 5.3. Glucose Oxidase Biosensors in Medicine Applications

#### 5.3.1. Cancer

The ability of GOx to consume intracellular glucose and oxygen to produce hydrogen peroxide and gluconic acid might allow it to be used in certain cancer therapy combinations. By consuming glucose, GOx could reduce the available metabolic energy sources of cancer cells, thereby, inhibiting their proliferation, and by consuming oxygen and producing gluconic acid, it could increase the hypoxia and acidity of the tumor microenvironment.

In several of these proposed combination treatments, GOx is embedded in nanocomposites. These come in a variety of forms, including hollow mesoporous silica nanoparticles, metal–organic frameworks, organic polymers, and magnetic nanoparticles are used for the construction of GOx-based nanocomposites for multi-modal synergistic cancer therapy [164].

In one proposed treatment [101], GOx chloroquine are attached to the surface and loaded into the cavity of rattle-structured polydopamine core-hollow mesoporous silica shell nanoparticles (PDA@hm). These nanoparticles are then used for a therapy combining energy metabolism regulation (by GOx), autophagy inhibition (by chloroquine) and low-temperature photothermal therapy (induced by the PDA nanocore). Photothermal therapy uses light-absorbing materials to convert photoenergy into local hyperthermia to destroy cancerous tissues [165,166].

In this treatment, GOx served to starve the tumor and directly suppressed the expression of the heat-shock proteins HSP70 and HSP90. In another proposed treatment [99], GOx–MnO${}_{2}$ nanosheets were developed to destroy cancer cells using a combination of starvation and self-oxygenation by GOx and photothermal therapy by the MnO${}_{2}$ nanosheet. In these nanostructures, the GOx catalytic activity could be enhanced by the hyperthermia triggered by near-infrared laser radiation.

These GOx–MnO${}_{2}$ structures exhibited pH and glucose responsive performance, activated by magnetic resonance and photoacoustic dual-modal imaging. Finally, GOx formed one component of a recently-designed “nanoreactor” consisting of a tirapazamine (TPZ)–human serum albumin–GOx mixture combined with a metal–polyphenol network consisting of Fe${}^{3+}$ ions and tannic acid [109].

This complex kills cancer cells by producing HO${}^{•}$, a process termed chemodynamic therapy (CDT) and TPZ${}^{•}$ radicals. The HO${}^{•}$ radicals are produced from H${}_{2}$O${}_{2}$ by Fe${}^{2+}$/Fe${}^{3+}$ ions through the Fenton reaction [167], while TPZ develops into a toxic radical under conditions of tumor hypoxia. The role of GOx in this assembly is to consume oxygen to increase the tumor’s hypoxia level, to produce H${}_{2}$O${}_{2}$ for conversion to HO${}^{•}$ and to consume glucose for starvation therapy.

#### 5.3.2. Diabetes Treatment

The earliest suggestion for a glucose sensor involving GOx was in 1962 [168]. Today, GOx is extensively used in the most common methods for measuring blood glucose levels [169,170]. Here, we limit ourselves to some relatively recent developments. GOx activity is highly oxygen dependent, which can lead to inaccuracies in amperometric β-d-glucose determinations.

Fokkert et al. [171] investigated the performance of fluorescence sensor-based and GOx-based glucose measurement during intensive exercise, when oxygen consumption is expected to be higher, and normal daily activities. They found that both methods were less accurate during exercise than during daily activities, and this finding persisted over the whole range of glucose concentrations examined.

Gutierrez et al. [172] attempted to overcome this problem by engineering GOx variants with lower oxygen dependency through random mutagenesis using error-prone PCR and sequence saturation. They discovered which positions seemed to be vital for oxygen sensitivity and for oxygen activity. One of their variants had a 37-fold reduced oxygen dependency but maintained the same β-d-glucose specificity and thermal resistance.

Other studies have attempted to use GOx to measure effective blood glucose levels non-invasively by monitoring other body fluids. Thus, Mohammadnejad et al. [106] developed a more precise, accurate and rapid method to measure blood glucose using GOx and peroxidase to oxidize a 4-[(hydroxy-3-methoxyphenyl)-azo]-benzenesulfonic acid (GASA) substrate. The stability of GASA and its oxidized products along with its direct and fast consumption by peroxidase, not only made it possible to determine blood glucose concentration with high reproducibility but also allowed it to be used for salivary samples.

A low-cost non-invasive paper-based biosensor for glucose measurements from tears has also recently been developed [94]. Finally, to eliminate some limitations and errors in some of the more commonly used monitoring systems, Jędrzak et al. [97] developed a biosensor consisting of magnetite, lignin and polydopamine bound to GOx together with ferrocene and a dedicated carbon paste electrode. The results showed that this biosensor has a potential for application in the determination of glucose in various commercial products.

One of the more interesting developments in the use of GOx in glucose monitoring is its potential to be coupled to an insulin delivery system, which could improve the health and quality of life for many diabetics. Yang et al. [173] developed a glucose and magnetic-responsive microvesicle delivery system, which can both regulate glucose levels and generate nitric oxide.

The injectable microvesicles are loaded with GOx, which can reduce hyperglycemia by consuming excess glucose. An applied magnetic field can then alter the permeability of the microvesicle shell, allowing the H${}_{2}$O${}_{2}$ produced by GOx to react with l-Arginine within the vesicle to produce nitric oxide, which has been shown to be important in the early stages of glucose-stimulated insulin secretion [174].

A glucose-responsive “closed-loop” insulin delivery system mimicking the function of pancreatic cells has potential to improve quality of life and health in diabetics. Yu et al. [107] created a glucose-responsive insulin delivery “closed-loop” system using a painless microneedle-array patch containing glucose responsive vesicles that are loaded with insulin and GOx. Under conditions of hyperglycemia, the GOx consumes glucose and oxygen, creating hypoxic conditions, which then trigger the release of the insulin.

This device effectively regulated the blood glucose in a mouse model of chemically induced type 1 diabetes. In a later refinement, the same group made an enhanced system where insulin release contingent on both hypoxia and H${}_{2}$O${}_{2}$ [108]. This system could effectively regulate blood glucose in mice with chemically-induced type 1 diabetes for 10 h.

Several glucose-responsive nanoparticles are based on phenylboronic acid, which has better stability compared with protein-comprised systems [175]. Chai et al. [100] created a glucose-responsive insulin delivery system comprised of poly(acrylamido phenylboronic acid)/sodium alginate nanoparticles loaded with GOx. The GOx-laded nanoparticles showed greater glucose sensitivity and faster glucose-responsive insulin release than nanoparticles loaded with insulin alone. These nanoparticles can also be easily prepared and have good biocompatibility.

### 5.4. GOx in Wound Healing: From Bench to Bedside

As mentioned above, GOx has several important applications in the food and medical industries. GOx’s ability to continuously produce hydrogen peroxide at low concentrations has attracted a great attention in the area of wound management. One wound treatment that makes use of the GOx-mediated release of hydrogen peroxide involves honey. Honey has been used for the treatment of a wide range of injuries, including acute and chronic wounds and burns [176].

A plethora studies have provided compelling evidence that hydrogen peroxide is the major antibacterial compound found in diluted honey. GOx, likely together with some polyphenols found in honey, is primarily responsible for the hydrogen peroxide generated during nectar processing and honey ripening. GOx, together with other enzymes catalyzing the metabolism of sugar, is produced by the hypopharyngeal glands of worker bees and secreted into honey [177].

Therefore, GOx is a regular but quantitatively variable compound in natural honey [178]. The final amount of hydrogen peroxide in dilute honey is a result of the enzymatic activity of both GOx and pollen-derived catalase enzymes. Interestingly, honeydew honeys, which are rich in polyphenols, are able to generate a higher level of hydrogen peroxide than many other honey types. It is believed that polyphenols, including flavonoids, might significantly contribute to higher levels of hydrogen peroxide [179].

The efficacy of the topical application of honey in the treatment of various wounds has been well-documented enough to allow it to be registered as a medical device in wound care management. GOx itself has become an interesting therapeutic platform in wound care [180,181,182].

There are several clinically tested GOx-based products available in the market for wound application containing a GOx enzyme immobilized in a suitable carrier (e.g., a hydrogel), which gradually releases a controlled amount of hydrogen peroxide [183,184]. However, a GOx-embedded hydrogel does not perfectly replicate antimicrobial honey-mimetic mechanisms because glucose is not added into the hydrogel.

Furthermore, the high concentration of H${}_{2}$O${}_{2}$ in the hydrogel could damage the normal tissues around the wound site. Most importantly, reactive oxidative species (ROS), such as HO${}^{•}$ are more effective in inhibiting and killing bacteria than H${}_{2}$O${}_{2}$. A variety of nanomaterials exhibiting the enzyme-mimicking activity (called nanozymes) are able to convert H${}_{2}$O${}_{2}$ into ROS species.

The development of nanozymes, such as metal-based nanoparticles and carbon-metal hybrid nanomaterials, which can simultaneously exhibit dual or multienzyme mimetic activity, are a promising antibacterial therapy in wound care.

Nanozymes with embedded GOx have very recently become the object of several studies investigating their wound-healing properties [185,186,187]. In a study by Du et al. [187], a nanozyme comprised of clinically approved iron oxide nanoparticles coated with GOx exhibited GOx, catalase and peroxidase-like activities.

Interestingly, in neutral and acidic microenvironments, it exhibited pH-switchable GOx/peroxidase and GOx/catalase cascade reactions, respectively. Thus, a fabricated Fe${}_{3}$O${}_{4}$–GOx nanozyme can, on the one hand, eradicate bacterial biofilm and shorten the inflammatory phase of wound healing and, on the other hand, accelerate the epithelialization and remodeling phase of wound healing. In addition, in vivo testing reveals that the Fe${}_{3}$O${}_{4}$–GOx nanozyme had superior healing activities against diabetic wounds caused by methicillin-resistant *Staphylococcus aureus* compared to unjoined Fe${}_{3}$O${}_{4}$ nanoparticles and GOx.

Although these nanozymes show effective antibacterial and wound healing properties, their direct use in clinical settings is considered questionable. Their unique characteristics, including their small size, chemical composition and solubility, may create a high risk and a hazard for human health. To address the limits of nanozymes in wound care, nanozymes can be incorporated into suitable wound dressings [188].

Zhang and co-workers [186] developed a novel wound dressing prepared from a nanozyme constructed by assembling GOx onto a hollow mesoporous carbon nanosphere doped with single-atom Fe and bacterial cellulose enveloped polypropylene composites. This nanozyme-based wound dressing exhibited outstanding breathability, biocompatibility and water uptake and antibacterial and antibiofilm ability.

## 6. Conclusions

Glucose oxidase catalyzes the oxidation of β-d-glucose to d-glucono-δ-lactone, which spontaneously hydrolyzes to d-gluconic acid and hydrogen peroxide in the presence of molecular oxygen. Due to its fast turnover and high stability and specificity, glucose oxidase has found many uses in industry and medicine.

Uses have been found for all aspects of its reaction: its consumption of glucose, production of H${}_{2}$O${}_{2}$, consumption of oxygen and production of gluconic acid have all found practical applications. Glucose oxidase is among the earliest of the enzymes to be used in this way, and new applications continue to be found.

Although the biochemical and biophysical characteristics of GOx have been thoroughly studied, there are still areas where additional study would be beneficial. For example, all but one of the structures of GOx presently known are variations of *A. niger* GOx. Although a number of industrial strains with enhanced stability or activity have been prepared, there is no structural analysis of what makes these forms more stable. Despite numerous efforts, there is still no structure of a GOx–substrate complex.

Moreover, some uncertainty still remains as to whether GOx might also be found in some bacteria or whether animals other than insects might produce it. Industrial research is also expected to develop new materials for biosensors and nanosensors and new ways of attaching GOx to them. In addition, GOx represents a promising molecule, which, in the form of nanozymes incorporated into wound dressings, may be of great use in wound care.

## Figures and Tables

**Table 1:** Tertiary structures of glucose oxidase determined by X-ray crystallography.

PDB ID	Resolution (Å)	Source	Reference	Remarks
1gal	2.3	*A. niger*	[9]	The earliest and lowest resolution structure.
1cf3	1.9	*A. niger*	[10]	
1gpe	1.8	*P. amagasakiense*	[10]	The only structure with a complete dimer in the asymmetric unit.
3qvp	1.2	*A. niger*	[11]	The highest resolution structure available.
3qvr	1.3	*A. niger*	[11]	
5nit	1.9	*A. niger*	[12]	A2 Mutant engineered for higher stability and turnover: T30V, I94V, A162T, R537K, M566V.
5niw	1.8	*A. niger*	[12]	A9 mutant: T30V, R37K, I94V, V106I, A162T, M566V.

**Table 2:** Proteins from the PDB similar in structure to *A. niger* glucose oxidase. Structures were identified by a BLAST search against the PDB using the *A. niger* sequence. Listed structures covered at least 75% of the query sequence and represent unique structures. All these proteins are members of the GMC oxidoreductase family.

PDB ID	RMSD	Description	Identity (%)	Reference
6xut	1.2704	*Trametes cinnabarina* Oligosaccharide dehydrogenase	35.89	[13]
4ynt	1.2841	*Aspergillus flavus* FAD glucose dehydrogenase	35.69	[14]
6zh7	1.4654	*Chlorella variabilis* Fatty acid Photodecarboxylase	27.99	[15]
5oc1	1.5672	*Pleurotus Eryngii* aryl-alcohol oxidase	29.67	[16]
6ze2	1.5827	*Chaetomium thermophilum* FAD-dependent oxidoreductase	31.24	[17]
4h7u	1.6328	*Agaricus meleagris* pyranose dehydrogenase	28.60	[18]
5hsa	1.6562	*Pichia pastoris* Alcohol Oxidase	23.56	[19]
6h3g	1.6725	*Phanerodontia chrysosporium* Alcohol oxidase	24.81	[20]
3nne	1.7055	*Arthrobacter globiformis* choline oxidase	26.31	[21]
4ha6	1.7182	*Mesorhizobium loti* pyridoxine 4-oxidase	26.85	[22]
6f97	1.7508	*Methylovorus* sp. 5-(hydroxymethyl)furfural oxidase	28.52	[23]
3q9t	1.7866	*Aspergillus oryzae* formate oxidase	25.55	[24]
6o9n	2.1470	*Myceliophthora thermophila* aryl-alcohol oxidase	30.75	[25]

**Table 3:** Some glucose oxidase substrates.

Substrate	GOx Activity (%) ^1^	Reference
β-d-glucose	100	[35,36,37,38]
2-deoxy-d-glucose	25–30	[36,37,38]
4-*O*-methyl-d-glucose	15	[37]
6-deoxy-d-glucose	10	[37]
4-deoxy-d-glucose	2	[37]
2-deoxy-6-fluoro-d-glucose	1.85	[38]
3,6-methyl-d-glucose	1.85	[38]
4,6-dimethyl-d-glucose	1.22	[38]
3-deoxy-d-glucose	1	[37]
6-*O*-methyl-d-glucose	1	[37]
α-d-glucose	0.64	[37,38]
mannose	0.2; 1	[36,37,38]
altrose	0.16	[37,38]
galactose	0.08	[36,37,38]
xylose	0.03	[36,37,38]
idose	0.02	[37,38]

^1^ Percent relative to β-d-glucose.

**Table 4:** GOx-producing organisms in at least one major database or at least one reference.

Organism Name	Accession or Reference ^1^	Identity ^2^
Fungi		
*Alternaria alternata*	[66]	–
*Aspergillus carbonarius*	U V9SH09	85.79
*Aspergillus niger*	U P13006 [3,55]	100.00
*Aureobasidium pullulans*	U A0A221SAG9	34.09
*Aureobasidium* sp.	U A0A1V0E5A9	31.69
*Ceratocystis fimbriata*	U A0A0F8CXS8	28.28
*Cladosporium neopsychrotolerans*	U A0A5Q2UVJ5, U A0A5Q2USS5	31.80, 32.56
*Cystobasidium laryngis*	[67]	–
*Dioszegia* sp.	[67]	–
*Flavodon flavus*	[68]	–
*Fusarium oxysporum*	[69]	–
*Goffeauzyma gastrica*	[67]	–
*Goffeauzyma gilvescens*	[67]	–
*Leucosporidium fragarium*	[67]	–
*Leucosporidium creatinivorum*	[67]	–
*Malassezia restricta*	U A0A3G2S2X3, U A0A3G2SBT7	29.44, 32.64
*Mucor circinelloides*	[70]	–
*Penicillium adametzii*	U A2I7K9 [71]	64.13
*Penicillium amagasakiense*	U P81156 [72]	65.74
*Penicillium canescens*	[73]	–
*Penicillium chrysogenum*	U K9L4P7 [74]	62.91
*Penicillium expansum*	[75]	–
*Penicillium janthinellum*	[76,77]	–
*Penicillium viticola*	U A0A0Y0IDS5	63.00
*Penicillium* sp.	U A0A7L7T1A0	65.51
*Penicillium* sp. RFL-2021a	N KAF7733001	34.33
*Phanerochaete chrysosporium*	[78]	–
*Pleurotus ostreatus*	[79]	–
*Pycnoporus cinnabarinus*	[80]	–
*Rasamsonia emersonii*	U A0A0F4YPS7	34.78
*Rhizopus stolonifer*	[81]	–
*Schizophyllum commune*	U D8QJE7 [82]	34.31
*Serendiptia indica* N CAG7851011		33.56
*Sporidiobolus salmonicolor*	[67]	–
*Talaromyces flavus*	U Q92452 [83]	63.97
*Talaromyces funiculosus*	[84]	–
*Talaromyces pinophilus*	[85]	–
*Talaromyces purpureogenus*	[86]	–
*Talaromyces stipitatus*	U B8MDS4 [87]	63.85
*Talaromyces variabilis*	U Q70FC9 [88]	66.61
*Thanatephorus cucumeris*	U M5BNG8 [89]	30.98
*Wickerhamomyces anomalus*	[67]	–
*Xylona heveae*	[90]	–
*Yarrowia* sp. B02	N KAG5360348	26.87
Insects		
*Apis melifera*	U Q9U8X6 [63]	26.66
*Helicoverpa armigera*	U B2MW81 [64]	29.40
*Heliothis viriplaca*	U A0A142I707	29.27
*Mythimna separata*	U A0A218N0E8	28.07
*Spodoptera exigua*	U D9ZFI1 [65]	28.74

^1^ Key: N—NCBI Protein, U—UniprotKB followed by accession number. ^2^ Relative to the *A. niger* GOx sequence (U P13006). – indicates no sequence available.

## Abbreviations

The following abbreviations are used in this manuscript:

GOx	Glucose Oxidase
FAD	Flavin Adenine Dinucleotide
NAD(P)	Nicotinamide Adenine Dinucleotide (Phosphate)
PQQ	PyrroloQuinoline Quinone
GMC	Glucose-Methanol-Choline
RMSD	Root-Mean-Squared Deviation
NMR	Nuclear Magnetic Resonance
NCBI	National Center for Biotechnology Information
SSF	Solid State Fermentation
PDA	PolyDopAmine
HSP70/90	Heat-Shock Protein 70/90
TPZ	Tirapazamine
CDT	ChemoDynamic Therapy
GASA	4-[(hydroxy-3-methoxyphenyl)-azo]-benzenesulfonic acid
ROS	Reactive Oxygen Species
